# Digital manufacturing techniques and the in vitro biocompatibility of acrylic-based occlusal device materials

**DOI:** 10.1007/s00784-024-05707-1

**Published:** 2024-05-15

**Authors:** Ketil Hegerstrøm Haugli, Dimitri Alkarra, Jan T. Samuelsen

**Affiliations:** 1 NIOM, Nordic Institute of Dental Materials, Oslo, Norway; 2 Dental Technology Program, Faculty of Health Sciences, Oslo Metropolitan University (OsloMet), OsloMet Box 4, St. Olavs plass, Oslo, 0130 Norway

**Keywords:** Resin, Occlusal device, Splints, Biocompatibility, Digital manufacturing, 3D print

## Abstract

**Objectives:**

Material chemistry and workflow variables associated with the fabrication of dental devices may affect the biocompatibility of the dental devices. The purpose of this study was to compare digital and conventional workflow procedures in the manufacturing of acrylic-based occlusal devices by assessing the cytotoxic potential of leakage products.

**Methods:**

Specimens were manufactured by 3D printing (stereolithography and digital light processing), milling, and autopolymerization. Print specimens were also subjected to different post-curing methods. To assess biocompatibility, a human tongue epithelial cell line was exposed to material-based extracts. Cell viability was measured by MTT assay while Western blot assessed the expression level of selected cytoprotective proteins.

**Results:**

Extracts from the Splint 2.0 material printed with DLP technology and post-cured with the Asiga Flash showed the clearest loss of cell viability. The milled and autopolymerized materials also showed a significant reduction in cell viability. However, by storing the autopolymerized material in dH_2_O for 12 h, no significant viability loss was observed. Increased levels of cytoprotective proteins were seen in cells exposed to extracts from the print materials and the autopolymerized material. Similarly to the effect on viability loss, storing the autopolymerized material in dH_2_O for 12 h reduced this effect.

**Conclusions/Clinical relevance:**

Based on the biocompatibility assessments, clinical outcomes of acrylic-based occlusal device materials may be affected by the choice of manufacturing technique and workflow procedures.

## Introduction

No other class of biomaterials has provided the dental clinician with such a wide range of applications as acrylic-based resins. Composite fillings, temporary restorations, fissure sealants, adhesive cement, removable dentures, and occlusal devices are some examples of dental biomaterials containing acrylic resin [1]. Occlusal devices are regularly used by patients in the treatment of different temporomandibular disorders (TMDs) or to inhibit the progression of dental wear from bruxism [2]. The production of occlusal devices has traditionally been performed with manual time-consuming heat-polymerization and autopolymerization techniques which has led to a demand for more cost-effective production. In recent years, digital solutions have made a significant impact on the selection and availability of materials and equipment in prosthetic dentistry. Cone beam computer tomography (CBCT) systems, intraoral scanners, and computer-aided design/computer-aided manufacturing (CAD/CAM) systems are to a great extent replacing traditional workflow procedures [3, 4]. In general, digital manufacturing of dental devices is conducted either through additive manufacturing (AM), commonly known as Three-dimensional (3D) printing, or subtractive manufacturing (SM), commonly referred to as milling [5]. The proposed advantages of using digital manufacturing techniques are reduced production time and increased product quality partly because of a decrease in human error from automation [5, 6]. Although milling has been the “gold standard” in CAD/CAM production, printing has become a feasible production alternative which might be more energy and time efficient, produce less material waste, and create geometries not possible with burs [5, 7].

Acrylic-based milling blocks are industrially pre-polymerized. Following standardized processing protocols, the companies can produce such blocks under optimal and controlled polymerization conditions [8]. The milling production of occlusal devices is generated by computer numeric control (CNC) milling units. Initially, the milling software calculates the milling operations and the selection of required burs based on the geometry of the imported virtual design. Then, the block is fixed in the milling machine and the milling operation is permitted.

Printing of acrylic-based occlusal devices is mainly utilized through vat photopolymerization [9]. This involves the shaping of the virtual design by curing liquid photopolymer resin in a tray onto a build platform. The two most common vat photopolymerization print technologies are stereolithography (SLA) and digital light processing (DLP) [10]. The main technological difference is that SLA cures each resin layer in the X/Y cross-section plane with a laser spot running point to point to form each layer, while the DLP technology projects a “mask of light” on the X/Y plane instantly controlled by a digital micromirror device (DMD) [11]. After printing, a wash in a solvent medium is performed to remove excess resin on the surfaces of the prints. Further, an additional post-curing procedure is required for printed acrylic-based materials intended for medical use aiming to enhance polymerization. Several post-curing units, protocols, and recommendations exist for this crucial procedure, raising questions regarding the efficacy and performance of these treatments on the biocompatibility of the materials.

The biocompatibility of a dental material depends on an appropriate host response when placed in the alternating harsh biological and physicochemical oral environment [12]. Acrylic-based devices consist mainly of different methacrylate monomers aiming to polymerize during the curing of the material. “The degree of conversion” (DC) is a measure of monomeric carbon-carbon double bonds converting into polymeric carbon-carbon single bonds and the DC varies among different polymer types and processing treatments [13]. Acrylic-based dental devices are known to release un-polymerized methacrylate monomers into the oral cavity which have been extensively investigated due to reported adverse effects from such monomers. Skin irritations and hypersensitivity reactions such as allergy are common adverse effects [14]. Studies on methacrylate-exposed cell cultures report increased oxidative stress, glutathione (GSH) depletion, cell growth disturbances, genotoxicity, and even cell death [14–16]. To counteract such stress, cells induce various cytoprotective mechanisms. A key cytoprotective mechanism involves the activation of the Nrf2/ARE (Nuclear factor erythroid 2-related factor/Antioxidant Response Element) signaling pathway [17, 18]. The pathway is involved in the regulation of many important antioxidant-related and detoxification genes such as glutamate-cysteine ligase (GCL; rate-limiting enzyme in the synthesis of GSH), p62, Hemeoxigenase-1 (HO-1) and glutathione-S-transferase [17, 19]. GSH protects cell components and structures from damage by the conjugation of methacrylates to GSH. The methacrylate-GSH adduct formation results in GSH depletion which is believed to cause increased reactive oxygen species (ROS) levels [20]. Redox imbalance and inefficient elimination of ROS, i.e. by GSH depletion, disrupt cellular homeostasis facilitating a risk of cytotoxicity and reduced viability [21].

There is a limited number of studies exploring the chemical composition and biocompatibility of novel CAD/CAM-produced acrylic-based resins [22, 23]. In the current study, the aim was to assess the biocompatibility of acrylic-based occlusal device materials produced from different digital manufacturing techniques. In vitro assessment of cell viability and the level changes of selected proteins will be used as a measure of cytotoxic potential.

## Materials and methods

### Overview of the selected materials and production workflows

A brief overview of the materials and the chemical composition given by the manufacturers are presented in Table 1. The production workflows are given in Table 2.

**Table 1:** Information on the selected materials and the chemical composition as given by the manufacturers

MATERIAL, MANUFACTURER AND LOT	COMPOSITION AS STATED IN MATERIAL DATA SAFETY SHEET (quantity in % if stated by the manufacturer)
**FREEPRINT Splint 2.0** DETAX, Ettlingen, Germany LOT: 220,807	Isopropylidenediphenol peg-2 dimethacrylate (Bis-EMA) (90 - < 95) 2-Propanoic acid, (5-ethyl-1,3-dioxan-5-yl)methyl ester (1 - < 5) diphenyl(2,4,6-trimethylbenzoyl)phosphine oxide (1 - < 5)
**Dental LT Clear V1** Formlabs, Somerville, USA LOT: XG461N03	Methacrylic oligomer (> 70) Glycol Methacrylate (> 20) Pentamethyl-piperidyl sebacate (< 5) Phosphine oxide (< 2.5)
**Therapon Transpa** Zirkonzahn, Gais, Italy LOT: 14,058	Acrylic polymethylmethacrylate copolymer.
**PalaXtreme** Kulzer, Hanau, Germany LOT: K010021 Powder Liquid	Only dangerous components documented 1-Benzyl-5-phenylbarbituric acid (0–5) Methyl methacrylate (≥ 0.1 - <1) 2-ethylhexyl thioglycolate (≥ 0.1 - <1) Dibenzoyl peroxide(≥ 0.1 - <1) Methyl methacrylate (75–90%) 2-[[(butylamino)carbonyl]oxy]ethyl acrylate (5–10%) Aliphatic urethane acrylate (0–5%) (2,4,6-trioxo-1,3,5-triazinane-1,3,5-triyl) triethylene triacrylate (≥ 1 - < 2.5) Quaternary ammonium compounds, tri-C8-10-alkylmethyl, chlorides (≥ 0.025 < 0.25)

**Table 2:** Information on the selected materials, production method, and post-processing treatments. The rows depict the seven workflows in the study design: Four for printing, one for milling, and two for autopolymerization

MATERIAL	PRODUCTION LINE (*n* = 7)	POST-PROCESSING TREATMENTS					
		Rinse	UV-curing			Grind and polish	dH_2_O storage
			Asiga Flash (AF)	Form Cure (FC)	Otoflash G171 (OF)		
FREEPRINT Splint 2.0	Print (DLP)	Isopropanol. 2 × 3 min in ultrasonic bath	15 min x 2			Wet SiC paper (1200 + 4000) grit	
	Print (DLP)	Isopropanol. 2 × 3 min in ultrasonic bath			2000 flashes x 2 with nitrogen gas	Wet SiC paper (1200 + 4000) grit	
Dental LT Clear V1	Print (SLA)	FormWash. Isopropanol 20 min		20 min 80 °C		Wet SiC paper (1200 + 4000) grit	
	Print (SLA)	FormWash. Isopropanol 20 min			2000 flashes x 2 with nitrogen gas	Wet SiC paper (1200 + 4000) grit	
Therapon Transpa	Milling					Wet SiC paper (1200 + 4000) grit	
PalaXtreme	Autopolymerization					Wet SiC paper (1200 + 4000) grit	1 h
	Autopolymerization					Wet SiC paper (1200 + 4000) grit	12 h

### Manufacturing and preparation of specimens

All the specimens were disc-shaped and carefully prepared aiming for final dimensions d = 13.0 mm and h = 2.0 mm. The manufacturer’s prescriptions were followed. A qualified dental technician was responsible for the production of the test specimens.

#### Specimens for printing

The specimens were virtually designed and stored in Standard Tessellation Language (STL) file format using 3D Builder (ver. 18.0.1931.0; Microsoft Corp., Redmond, WA, USA). The virtual design was slightly larger (d = 13.05 and h = 2.05) to compensate for surface preparations to final dimensions. Further, the design was imported into the corresponding printer software (PreForm ver. 3.5.1; Formlabs, Sommerville, USA. Asiga Composer ver. 1.2.12; Asiga, Sydney, Australia). The specimen design was replicated and vertically orientated (90 ° angle relative to the build platform) and positioned in the center of the build platform. Supportive structures were placed on the sidewalls of each specimen to ensure that the supportive structures do not interfere with grinding and polishing of the larger flat surfaces. All specimens were printed using standard printing parameters.

Two commercially available occlusal splint materials for 3D printing (Dental LT Clear V1; Formlabs, Somerville, MA, USA. FREEPRINT Splint 2.0, Detax, Ettlingen, Germany) were selected. The Dental LT Clear material was printed with stereolithography (SLA) technology (Form 2; Formlabs, Sommerville, MA, USA), while the FREEPRINT Splint 2.0 material was printed with digital light processing (DLP) technology (Asiga MAX UV; Sydney, Australia). The layer thickness was set to the highest possible resolution for the materials i.e., 100 μm for Dental LT Clear V1 and 50 μm for the FREEPRINT Splint 2.0. Specimens were rinsed in 99.5% isopropyl alcohol. To ensure the elimination of excess alcohol on the surfaces, the specimens were air-dried for 30 min at room temperature. Post-curing was performed either with the manufacturer´s curing unit, Asiga Flash [AF] or Form Cure [FC], or the standalone curing unit Otoflash G171 (NK-Optik GmHb, Baierbrunn, Germany) [OF] with N_2_ inert gas.

#### Specimens for milling

Virtual cylinders were stored in STL file format using 3D Builder, imported into the milling unit (M1 Wet Heavy Milling unit; Zirkonzahn, Gais, Italy), and milled out of the resin block (Therapon Transpa, Zirkonzahn) using 2 L (Item number: FR6001, Zirkonzahn) and 1 L (Item number: FR6003, Zirkonzahn) PMMA burs. After milling, the cylinders were cut into discs to the desired dimensions under constant water cooling (Accutom 100 M; Struers, Ballerup, Denmark).

#### Specimens for autopolymerization

Printed discs were used as a replica to make a silicone matrix (Heraform; Kulzer, Hanau, Germany) for the pouring of the autopolymerization resin (PalaXtreme, Kulzer, Hanau, Germany). The powder-to-liquid ratio complied with the manufacturer’s recommendations and mixed accordingly. The liquid resin was poured into the silicone matrix and secured with a glass plate mounted on top before transferring the matrix to a pressure polymerization chamber (Polymax 5; Dreve Dentamid, Unna, Germany) curing the specimens at 55 °C for 40 min and 2 bar pressure. After curing, the grinding and finishing procedures were undertaken (see 2.2.4). Finally, the specimens were post-treated either by storage in distilled water (dH_2_O) at room temperature for 1 h or 12 h and grouped accordingly.

#### Grinding and finishing of the specimens

Supportive structures were carefully removed with a tungsten carbide bur. Specimen surfaces were shaped to desired dimensions by wet grinding with 1200 FEPA P silicon carbide discs (Struers, Ballerup, Denmark). A high gloss surface finish was achieved with wet polishing using 4000 FEPA P discs. A micrometer gauge was frequently used to measure the surfaces during the grinding and polishing protocol to confirm compliance with the final dimensions. All specimens were then ultrasonically cleaned in dH_2_O for 10 min and dried at room temperature for 15 min.

### Cell culture conditions and exposure studies

PE/CA-PJ49, a human oral squamous cell carcinoma cell line, was purchased from the European Collection of Authenticated Cell Cultures, (ECACC). To achieve a complete cell culture medium, Nutrient Mixture Kaighns Modification medium (F-12 K Nut Mix; Gibco, Life Technologies, CA, USA) was supplemented with 5% fetal bovine serum (FBS; Sigma-Aldrich, St. Louis, USA) and penicillin/streptomycin (100 units/ml / 100 µg/ml; Lonza, Verviers, Belgium). The cell line was grown in sterile flasks containing the complete medium and maintained in a humidified atmosphere at 37 °C and 5% CO_2_ (split to passage 5–6, confluence approximately 70–80%). Seeding of the cells was made on plates (Costar; Corning Inc., NY, USA) in a density of approximately 150.000 cells/ml for 24 h before exposure experiments.

To attain extracts, grouped specimens relative to the specified workflows were stored in 50 ml sealed tubes containing complete medium following the surface-area-to-volume ratio of 3 cm^2^/ml as given in ISO 10993-12 [24]. The tubes were maintained in a water bath (37 °C, 24 h) and subjected to repetitive sideways motions. Finally, the extracts were sterile filtered into new 50 ml tubes.

In the exposure studies, 100% extract was pure sterile filtered extract. A dilution series with the complete medium, treated in the same manner as the extracts, was used for extract dilution. To ensure assay validity for both the MTT assay and the Western blot immunoassay, the complete medium was provided as a negative control (blank) while 2 mM HEMA mixed with the complete medium served as a positive control.

### MTT assay as a measure of cell viability

The MTT (3-(4,5-dimethylthiazol-2-yl)-2,5-diphenyl tetrazolium bromide) assay was conducted mainly based on ISO 10993-5 [25]. The assay depends on the conversion of MTT to the dark blue, water-insoluble tetrazolium salt formazan by mitochondrial dehydrogenases. The number of viable cells correlates to the color intensity determined by photometric measurements. In brief, the cell culture medium was replaced with MTT solution (0.5 mg/mL MTT, Sigma-Aldrich, St Louis, MO, in PBS) in control and exposed cell culture. Following 1 h of incubation at 37 °C / 5% CO_2_, the MTT solution was removed. 500 µL DMSO (Sigma-Aldrich) was added to dissolve the formazan formed. The plate was shaken for 15 min and absorption at 570 nm was measured using a spectrophotometer (Synergy H1; BioTek Instruments, Winooski, VT).

### Western blot immunoassay to quantify protein levels

A list of the antibodies used in the assay is given in Table 3.

**Table 3:** List of antibodies used in the Western blot analysis

Antibody	Supplier ^a^	Product number
^1^ NAD(P)H quinone oxidoreductase 1 (NQO1)	CST	CS-3187
^1^ P62/SQSTM1	ProGen	GP62-C
^1^ Pirin	Abcam	ab157212
^1^ Hemeoxigenase 1 (HO-1)	Abcam	ab13243
^1^ Glutamate-cysteine ligase regulatory subunit (GCLC)	Thermo	PA5-19702
^2^ Guinea Pig IgG	LiCor	925-68077
^2^ Mouse IgG	LiCor	926-32210
^2^ Rabbit IgG	LiCor	926-32211
^3^ β-actin	Abcam	ab184092

^1^ Primary antibody

^2^ Secondary antibody

^3^ Loading control

^a^ CST: Cell Signaling Technology Inc. (Beverly, MA). Abcam: Abcam (Cambridge, UK), Thermo: Thermo Fisher Scientific (Waltham, MA), Progen: ProGen Biotechnik GmbH, (Heidelberg, Germany), LiCor: LI-COR Biotechnology (Lincoln, NE)

^a^ CST: Cell Signaling Technology Inc. (Beverly, MA). Abcam: Abcam (Cambridge, UK), Thermo: Thermo Fisher Scientific (Waltham, MA), Progen: ProGen Biotechnik GmbH, (Heidelberg, Germany), LiCor: LI-COR Biotechnology (Lincoln, NE).

The culture medium was removed, and the cells were washed three times in 1 x PBS. Then, 200 µl of SDS sample buffer (12% SDS, 30% glycerol, 150 mM Tris/HCl to reach pH = 7.0) was added to the plate for cell lysis. Further, the cells were scraped and stored in sealed Eppendorf tubes on the bench overnight. Sonication with a 3 mm ultrasonic probe, 25% amplification, and pulses at 7 × 5 s (Vibra-Cell VCX 130; Sonics, Newtown, CT) was performed. The probe was cleaned with dH_2_O between each lysate to be sonicated. 2-mercaptoethanol (Sigma-Aldrich) and bromophenol blue (Sigma-Aldrich) were added to each tube for S-S bond cleavage and staining, respectively, before loading and separation of proteins on a 10% sodium dodecyl sulfate-polyacrylamide gel by electrophoresis. The gel containing the separated proteins was blotted (transferred) onto nitrocellulose membranes. Staining with ponceau and beta-actin was used as loading controls. The membranes were blocked for 30 min with 3% bovine serum albumin (BSA; Sigma-Aldrich) in Tris-buffered saline (TBS) containing 0.1% Tween 20 (TBS-T). The selection of investigated proteins was mainly based on previous in vitro findings [26]. Primary antibodies targeting each of the proteins Glutamate cysteine ligase catalytic subunit (GCLC), p62, HO-1, Pirin, and NAD(P)H Quinone Dehydrogenase 1 (NQO1) were each diluted in TBS-T containing.

1% BSA and incubated on the membranes at 4 °C overnight on a shaker. Then, the membranes were washed three times in TBS before incubation of the membranes with secondary antibodies in TBS-T and 1% BSA for 2 h at room temperature. The immunoreactive protein of interest was detected and quantified using the Odyssey CLx imaging system (LI-COR Biotechnology, Lincoln, NE).

### Statistics

Raw data from MTT and Western blot analyses were normalized according to a two-step normalization protocol [27] before statistical analysis. In short, each value in an experimental replicate was divided by the mean of all the data in that experiment. Further, each value was normalized to the mean of all controls setting the average control to 100% while still maintaining variation in the control groups for the ANOVA test. The analyses were performed using statistical software (GraphPad Prism ver. 9.4.1, San Diego, CA). The normality was visually inspected by Q-Q plots and evaluated with the Shapiro-Wilk test. One-way ANOVA was used for the milling technology workflow, while two-way ANOVA was used for the 3D printing workflows and the autopolymerization workflow to compare the independent post-processing variables. The results are based on three to four replicates (*n* = 3–4) and presented as mean ± SD. Asterisk (*) indicates statistical significance (*p* < 0.05) relative to the control group.

## Results

Table 4 summarizes the two-way ANOVA results showing the effect of the two independent categorical variables (extract concentration and post-processing method) and the interaction term.

**Table 4:** Summary of the two-way ANOVA analysis

Categories	Interaction?	Sig. extract concentration	Sig. post-processing method
MTT – Splint 2.0	Yes ***	***	***
MTT – Dental LT Clear	No	*	ns
MTT – PalaXtreme	No	ns	***
Western – Splint 2.0 - GCLC	No	**	ns
Western – Splint 2.0 - P62	No	**	ns
Western – Splint 2.0 - Pirin	No	ns	ns
Western – Splint 2.0 - NQO1	No	ns	ns
Western – LT Clear - GCLC	No	*	ns
Western – LT Clear - P62	No	**	ns
Western – LT Clear - Pirin	No	ns	ns
Western – LT Clear - NQO1	No	**	ns
Western – PalaXtreme - GCLC	Yes **	**	***
Western – PalaXtreme - P62	No	ns	ns
Western – PalaXtreme - Pirin	No	**	**
Western – PalaXtreme - NQO1	Yes *	*	***

*p < 0.05, ** *p* < 0.01, *** *p* < 0.001. ns = not significant, i.e. *p* > 0.05

### MTT assay

**Fig. 1:**
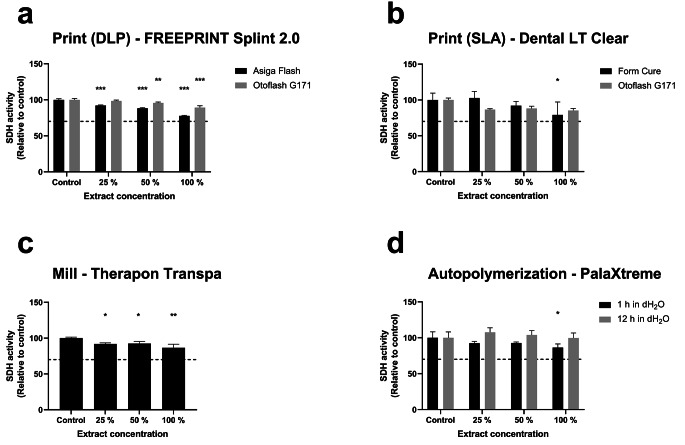
MTT results from the printing workflows (**a** and **b**), the milling workflow (**c**), and the autopolymerization workflows (**d**). The results reflect mitochondrial succinate dehydrogenase (SDH) activity in the cells after exposure to extract concentrations. The asterisk indicates a significant difference between the group relative to the control group of the specified post-treatment (* *p* < 0.05, ** *p* < 0.01, *** *p* < 0.001). The dashed line denotes the 70% threshold value for cytotoxic potential as given by ISO 10993-5

The MTT results for the selected materials and the manufacturing workflows are shown in Fig. 1.

The results indicate a significant decrease in cell viability relative to control for all manufacturing techniques except for the print material Dental LT Clear (STL) post-cured with OF, and the autopolymerization material immersed in dH_2_O for 12 h. The Splint 2.0 material (DLP) post-cured with AF showed the most significant reduction in cell viability among all extract concentrations while post-curing the material with OF showed the least reduction in cell viability among the print workflows although there were significant differences relative to control for 50% and 100% extract concentration. The mill material Therapon Transpa also showed a significant reduction in cell viability for increasing extract concentrations. For the autopolymerization material, the 12-hour dH_2_O storage group showed no alteration in cell viability relative to the control, while the 1-hour dH_2_O storage group showed a significant difference from the control at 100% concentration.

### Western blot

**Fig. 2:**
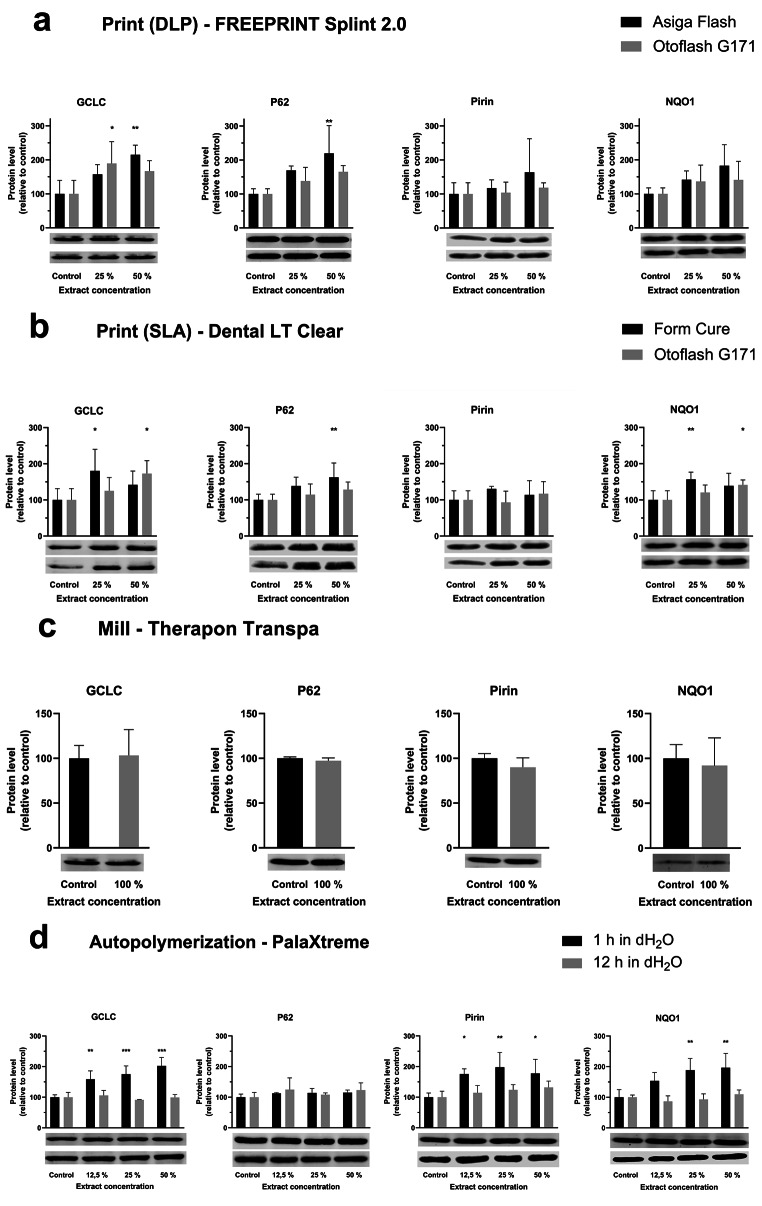
Western blot results from the printing workflows (**a** and **b**), the milling workflow (**c**), and the autopolymerization workflows (**d**). The Western blots show the protein levels of GCLC, P62, Pirin, and NQO1 after exposure of the cell line to material-based extracts representing the different manufacturing workflows (**a**, **b**, and **d** have two blot panels; the upper panel denotes the black bars while the lower panel denotes the grey bars). The asterisk indicates a significant difference between a group relative to the control of that group (* *p* < 0.05, ** *p* < 0.01, *** *p* < 0.001)

The Western blot results (Fig. 2) show the exposure scenarios with extract concentrations and the workflows relative to the protein of interest.

The positive control (2 mM HEMA) verified the detection of the target proteins (not shown). The HO-1 protein was not detected in any of the manufacturing workflows. For the print materials, increased levels of the proteins GCLC, P62, and NQO1 were observed. No clear difference between the post-curing method relative to protein expression was observed. No alterations in protein levels among the selected proteins were shown for the milling material even at 100% extract. For the autopolymerization material, the workflow representing the 12-hour dH_2_O storage showed no significant alterations in protein levels for all extract concentrations, while the 1-hour dH_2_O storage workflow presented increased protein levels of GCLC, Pirin, and NQO1.

## Discussion

The use of CAD/CAM systems for planning, design, and production in dentistry provides many benefits for both healthcare professionals and patients. However, the rapid implementation of such systems and associated materials makes it challenging to ensure that patients are treated with optimal oral devices in terms of biocompatibility [28]. Research focusing on the safe use of CAD/CAM biomaterials is therefore vital.

Adverse reactions from biomaterials depend on the patient being exposed to components released from the biomaterial. It should be emphasized that the polymerization process from acrylic-based biomaterials never reaches completeness which facilitates the release of uncured leakage products from the materials regardless of the manufacturing technique. Such leakage products are commonly residual acrylate monomers and other chemical additives [29]. The concentration of the leakage products mainly depends on factors such as polymerization type, polymerization time, and polymerization temperature [30]. CAD/CAM production introduces processing variables that may affect the biocompatibility of the biomaterials [31, 32]. The milling production has less processing variables compared to the printing counterpart since the material is pre-polymerized, and the post-processing only involves grinding and polishing. The acrylic-based milling blocks are produced under high temperature and pressure to optimize the polymerization process and achieve a higher DC [33, 34]. However, long production time and material waste from ineffective use of the material block are drawbacks associated with milling. Printing overcomes most of these challenges, although more workflow parameters are imposed on the individual manufacturer of the dental device. The printing process entails the conversion of a liquid resin to a solid material: Different printing technologies, liquid resin chemistries, orientation of the print objects, and the print layer thickness are all sources of variation [35–38]. Further, post-processing steps such as isopropyl rinse and different post-curing methods are variables to be aware of [39]. In summary, high variation in the biocompatibility of printed devices may occur.

Therefore, the current study aimed to assess the impact of different manufacturing workflows and associated post-processing variables and their effect on material biocompatibility. In vitro cell viability and levels of cytoprotective proteins were chosen as parameters to measure biocompatibility [26].

### MTT cell viability

The MTT assay results showed a significant decrease in cell viability among several manufacturing techniques and workflows (Fig. 1) compared to unexposed controls. Nevertheless, all the materials tested showed above 70% cell viability at all extract dilutions. According to ISO 10993-5, a sample is considered to have a cytotoxic potential if the viability is reduced to less than 70% [25]. It must be emphasized, however, that this viability threshold cannot decisively be regarded as proof for not being cytotoxic, i.e., lack of evidence on cytotoxic response is not evidence of the opposite. Although the MTT assay is widely used, the MTT results rely entirely on the assumption that mitochondrial dehydrogenase activity is constant in all viable cells. Even though this should be fulfilled, the assay cannot discriminate between cell death and cell growth inhibition [40]. Also, cell viability results from monomer exposure have shown to be largely dependent on the cell model systems used [41]. In addition, interlaboratory tests have shown that the ISO 10993-5 specifications set are not explicit enough to obtain comparable results for a medical device [42]. In summary, misinterpretations, overshadowing of possible cytotoxic effects, and false conclusions could be the result. This also highlights the caution that must be taken when in vitro results are used in the translation to clinical settings. The conduction of additional biological assays is, therefore, highly recommended to support MTT results.

In our study, the Splint 2.0 material (DLP) post-cured with AF demonstrated a significant decrease in viability for all treatment groups. Reymus et al. [10] observed that the choice of post-curing method had an impact on the DC of printed occlusal device materials concluding that specimens post-cured with OF achieved the highest DC. Assuming that cell viability is correlated with DC, this is in line with our results for the DLP workflow. While the AF unit facilitates constant curing from 4 × 9 W UV lamps, the OF curing unit irradiates with two xenon lamps (200 W in total) flashing at 10 Hz in a wide spectrum range (280–700 nm). In addition, the unit facilitates N_2_ inert gas during post-curing to prevent the formation of an oxygen-inhibited layer [43]. The presence of this layer on the surface of the material will reduce the DC [44]. In clinical settings, higher levels of unpolymerized monomers, where the oxygen-inhibited layer was present, have also been reported [45]. Post-processing by grinding and polishing is effective in the removal of this unpolymerized layer [46], however, grinding and polishing of the interior parts of resin-based occlusal devices is contraindicated as this may affect the fitting of the device. In this respect, it has been reported significantly higher MMA concentrations in the salivary film on the fitting side of acrylic orthodontic appliances compared to whole saliva samples highlighting the clinical relevance of this issue [47]. In our study design, the specimens were ground and polished on all sides removing the inhibition layer. This ideal condition may facilitate possible underestimation of leakage products relative to clinical settings.

A reduction in cell viability at higher extract concentrations is evident for the Dental LT Clear material (SLA). No clear difference in cell viability is shown when comparing the OF and FC workflows. The FC unit irradiates with 13 LEDs giving a total of 39 W output. The LEDs emit close to UV (405 nm) which has the benefit of curing thicker parts more efficiently than light sources of lower wavelengths [48]. Although our study design cannot specifically evaluate the effect of temperature, elevated post-curing temperatures from the FC unit may increase reaction kinetics facilitating greater access of monomers to growing polymer chains. In other studies, the effect of increased post-curing temperature on acrylic-based resins has demonstrated an improvement in mechanical properties, degree of conversion, and biocompatibility [49, 50]. In a study by Jung-Hwa et al. [51] they found that increased temperature in combination with oxygen shielding (inert gas or glycerol) enhanced both the mechanical properties and the DC of acrylic-based prints.

The PMMA-based material for milling is assumed to be highly polymerized. However, the MTT results showed reduced cell viability in an extract concentration-dependent manner. In a study by Bürgers et al. [52], the measured cytotoxicity of the same material presented different cell viability results depending on the applied cell line, but the cell viability was not below 70%. Similarly, a slight cytotoxic effect was shown for PMMA interim material blocks [53]. The reduction in cell viability may be explained by the elution of other resin matrix additives (initiators, inhibitors, accelerators, plasticizers, etc.), however, this was not evaluated in our study.

The analyses of the autopolymerized material stored for one hour in dH_2_O showed a significant decrease in cell viability. In contrast, no viability loss was observed in the analyses of material stored for 12 h in dH_2_O. Up to 24 h of water storage has been suggested to be a standard procedure for all autopolymerized acrylic-based appliances before insertion into the patient´s mouth [47]. A study by Na-Eun [54] showed that immersing printed resins in 100 °C water for 5 min increased cytocompatibility and the DC. Although storing print objects in water as a post-processing procedure is not declared for any of the CAD/CAM materials, this might be beneficial in terms of material biocompatibility.

### Levels of cytoprotective proteins

To further assess possible cytotoxic responses, protein expression analysis was performed by standard Western blot immunoassay. Based on previous studies of HEMA-exposed cells [26, 55], five proteins (HO-1, Pirin, NQO-1, p62, and GCLC) were chosen. The proteins are vastly associated with cellular stress response and an up-regulation of the proteins can be interpreted as increased demand for cytoprotection. Becher et al. [26] used microarray to quantify RNA transcripts of the 25 most up-regulated genes when a BEAS-2B cell line was exposed to 2-hydroxyethyl methacrylate (HEMA). They found that HO-1 was most upregulated (4,44-fold change). In contrast, our study on PJ49 cells did not detect HO-1 from any of the exposure scenarios.

The Splint 2.0 material (DLP) showed a trend in the upregulation of the proteins GCLC, NQO1, and p62 in a concentration-dependent manner, although not significant. However, experiments using specimens post-cured with AF depicted a higher trend in the upregulation of the proteins compared to those post-cured with OF. This may support a higher DC in the materials post-cured with OF as upregulation of the proteins can be linked to increased release of electrophilic monomers [55]. Exposure to electrophilic monomers have previously been shown to cause GSH depletion and increased levels of oxidative stress [56]. Increased levels of GCLC and NQO1 can be a response to such events. The p62 protein is associated with autophagy. Increased autophagy improves cellular stress resistance since autophagy decreases metabolic load and toxicity by removing damaged cellular components [57]. Also, the cells exposed to Dental LT Clear (STL) extract showed upregulation of the cytoprotective proteins in a concentration-dependent manner, although not as prominent as the DLP workflow. Samuelsen et al. [55] observed an upregulation of Pirin in THP-1 cells exposed to 2 mM HEMA. In our study, we could not see any changes in Pirin levels regarding the print workflows.

Although the PMMA milling material showed some degree of cell viability reduction, no alterations in relative protein expression compared to the control were shown for the selected proteins at 100% extract concentration. Assuming that methacrylates in general activate the Nrf2 pathway, our results indicate that other leakage products are responsible for the observed viability loss. A study designed to determine such leakage products could potentially verify this hypothesis.

Exposure to autopolymerization extracts showed that specimens stored in dH_2_O for 12 h demonstrated a decrease in expression of Nrf2-associated proteins compared to 1-hour dH_2_O storage. Among all the workflows, Pirin is only significantly increased in the 1-hour dH_2_O storage group. Since the autopolymerization material is comprised mainly of MMA it might be assumed that MMA exposure to the cells increases Pirin expression. However, this can only be determined by analytical methods aiming to quantify and identify specific monomers or additives.

Much is known about the role of Nrf2 in detoxification and response to stress, however, the evidence on how Nrf2 regulates immune cell functions is less understood [58]. Nrf2 is believed to play a protective role in contact dermatitis by upregulation of antioxidant and cytoprotective genes. Increased mRNA levels of NQO1 and HO-1 have been reported in response to several contact sensitizers in dendritic cells and THP-1 monocytic cells [59]. Nrf2 has also been demonstrated to recruit neutrophils in contact hypersensitivity [60]. In the same study, they compared knock-out mice with wild-type mice and found an upregulation of Nrf2 antioxidant genes (HO-1, GCLC, and NQO1) supporting Nrf2´s critical role in skin hypersensitivity. In line with this, Nrf2 activity is used as a measure of sensitizing potential [61]. Hence, our results may indicate that printed acrylic-based materials, giving a higher upregulation of Nrf2-associated proteins, could result in a higher sensitization potential than milled acrylic-based materials. Also, water storage of acrylic-based dental devices for 12–24 h seems beneficial to reduce exposure to material leakage products. This may also reduce the possible risk of sensitization reactions from such leakage products.

This study has investigated the effect of some selected workflow variables. In printing, several other post-processing variables can potentially influence biocompatibility. Parameters such as printing orientation, print layer thickness, and rinsing solvents are some other parameters that could be more specifically considered in future studies. Grymak et al. [62] investigated the hardness and polishability of different occlusal device materials. They found that printed specimens treated with the same polishing protocol differed both in surface roughness and hardness depending on the chosen print angle. Such variations in surface characteristics may affect the in vitro biocompatibility of occlusal device materials. Although all specimens were visually confirmed in our study, only minor deviations between parallel specimens were shown in the MTT assay suggesting consistent results with the applied grinding and polishing protocol. In addition, using SiC paper discs for grinding and polishing is used for standardization purposes which deviates from clinical settings were tungsten carbide burs, rubber or cotton wheels with high gloss polishing paste are used. There is always a risk of contamining the materials from such procedures, while heat generated from high gloss polishing without water cooling might alter surface characteristics of the specimens. Such factors could have affected the source of variation when conducting the following biological assays in the current study. Other variables may also alter surface characteristics in a clinical setting. Greil et al. [63] demonstrated increased water sorption of printed denture base materials compared to other manufacturing techniques. Water sorption causes swelling and a decrease in hardness of the material which may further increase material abrasion accelerating the release of wear particles and leakage products.

Lastly, with emerging technologies and a rapid introduction to novel dental materials, a demand for more informative and accurate safety data sheets regarding the content of the materials should be encouraged. This will provide both the scientific society in the biomaterial field as well as the health profession with a better foundation in decisions on material biocompatibility and patient safety-related issues.

## Conclusion

In summary, our findings indicate that the choice of manufacturing technique and post-processing treatments of acrylic-based occlusal devices impact biocompatibility. Digitally manufactured occlusal device materials did present some degree of viability loss on the cells. The level of the selected cytoprotective proteins varied among the print materials. This effect was not observed for the milling material nor the autopolymerization 12-hour dH_2_O group. Prolonged water storage seems to reduce the in vitro toxicity.

## Data Availability

No datasets were generated or analysed during the current study.
